# Organotin Compound Derived from 3-Hydroxy-2-formylpyridine Semicarbazone: Synthesis, Crystal Structure, and Antiproliferative Activity

**DOI:** 10.1155/2010/718606

**Published:** 2010-05-12

**Authors:** Joanna Wiecek, Dimitra Kovala-Demertzi, Zbigniew Ciunik, Joanna Wietrzyk, Maria Zervou, Mavroudis A. Demertzis

**Affiliations:** ^1^Inorganic and Analytical Chemistry, Department of Chemistry, University of Ioannina, 45110 Ioannina, Greece; ^2^Faculty of Chemistry, University of Wrocław, 14 F. Joliot-Curie St., 50-383 Wrocław, Poland; ^3^Institute of Immunology and Experimental Therapy, Polish Academy of Sciences, 12 R Weigl St, 53-114 Wrocław, Poland; ^4^Laboratory of Molecular Analysis, Institute of Organic and Pharmaceutical Chemistry, National Hellenic Research Foundation, 48, Vas. Constantinou Ave, 11635 Athens, Greece

## Abstract

The novel diphenyltin(IV) compound [Ph_2_(HyFoSc)Sn] (**2**), where H_2_HyFoSc (**1**) is 3-hydroxy-2-formylpyridine semicarbazone, was prepared and characterized by vibrational and NMR (^1^H, ^13^C) spectroscopy. The structure of [Ph_2_(HyFoSc)Sn] was confirmed by single-crystal X-ray crystallography. The doubly deprotonated ligand is coordinated to the tin atom through the enolic-oxygen, the azomethine-nitrogen, and phenolic-oxygen, and so acts as an anionic tridentate ligand with the ONO donors. Two carbon atoms complete the fivefold coordination at the tin(IV) center. Intermolecular hydrogen bonding, C–H → *π*, and *π* → *π* interactions combine to stabilize the crystal structure. Compounds **1** and **2** have been evaluated for antiproliferative activity in vitro against the cells of three human tumor cell lines: MCF-7 (human breast cancer cell line), T24 (bladder cancer cell line), A549 (nonsmall cell lung carcinoma), and a mouse fibroblast L-929 cancer cell line.

## 1. Introduction

Organotin compounds are of interest in view of their considerable structural diversity [1]. Increasing interest in organotin(IV) chemistry has arisen in the last few decades and is attributed to their significantly important biological properties. Several di- and tri-organotin species have shown potential as antineoplastic and antituberculosis agents [2–5]. The binding ability of organotin compounds towards DNA depends on the coordination number and nature of groups bonded to the central tin atom. The phosphate group of DNA-sugar backbones usually acts as an anchoring site and DNA base-nitrogen binding is extremely effective and this often results in the stabilization of the octahedrally coordinated tin center. Recent studies have showed that low doses of organotins can exhibit antitumoral activity and have suggested a mode of action via a gene-mediated pathway in the cancer cells, opening a new research subarea on organotin compounds [6].

Thio- and semicarbazones (TSC) possess a wide range of bioactivities, and their chemistry and pharmacological applications have been extensively investigated. The more significant bioactivities of a variety of semicarbazones (antiprotozoa, anticonvulsant) and thiosemicarbazones (antibacterial, antifungal, antitumoral, antiviral) and their metal complexes have been reviewed together with proposed mechanisms of action and structure-activity relationships [7, 8]. Casas et al. [9] have surveyed structural aspects of main group metal complexes of semicarbazones and thiosemicarbazones. The survey shows that heterocyclic and nonheterocyclic TSC's are very versatile coordination agents with these elements [9].

Following our interest in the chemistry and pharmacological properties of thiosemicarbazones [10–17] and towards organotins [18–21], herein, the preparation and spectroscopic characterization of a novel semicarbazone and a novel diphenyl organotin compound derived from the reaction of SnPh_2_O with 3-hydroxy-2-formylpyridine semicarbazone H_2_HyFoSc (**1**) are described with the final goal of developing new biologically active pharmaceuticals. The results of the cytotoxic activity of **1**, SnPh_2_O, and of the organotin compound (**2**) against the cells of three human cancer cell lines: MCF-7 (human breast cancer cell line), T24 (bladder cancer cell line), A549 (non-small cell lung carcinoma), and a mouse fibroblast L-929 cancer cell line are also reported. To our knowledge, this is the first report of synthesis of **1 **and **2**.

## 2. Experimental

### 2.1. General and Instrumental

The reagents (Aldrich, Merck, Sigma) were used as supplied while the solvents were purified according to standard procedures. Melting points were determined in open capillaries and are uncorrected. Infrared and far-infrared spectra were recorded on a Perkin–Elmer Spectrum GX FT IR System spectrophotometer using KBr pellets (4000–400 cm^−1^) and nujol mulls dispersed between polyethylene disks (400–40 cm^−1^). The ^1^H, ^13^C NMR spectra were recorded on a Bruker AC-300 MHz and on a Varian 600 MHz spectrometer. The spectra were acquired at room temperature (298 K). The chemical shifts are reported in ppm with respect to the references (external tetramethylsilane (TMS) for ^1^H and ^13^C NMR). Elemental analyses were carried out by the microanalytical service of the University of Ioannina, Greece.

### 2.2. Synthesis

#### 2.2.1. 3-Hydroxypyridine-2-carbaldehyde Semicarbazone (**1**)

 Commercially available 3-hydroxy-(2-hydroxymethyl)pyridine hydrochloride was oxidized with MnO_2_, prepared by heating MnCO_3_ for 12 h at 300°C, according to [22, 23] to afford 3-hydroxypyridine-2-carbaldehyde as a yellow powder, yield 62%, and m.p. 77°C. The aldehyde (2 mmol) in EtOH (6 mL) was then reacted with a solution of semicarbazide hydrochloride (2 mmol) in H_2_O (3 mL) at 80°C for 2 h. Then, the mixture was kept in a refrigerator overnight. The resulting yellow powder was filtered off and recrystallized from EtOH. The powder was washed with cold EtOH and dried in vacuo over silica gel at 40–50°C for 4 h to afford **1** as a yellow powder, yield 75%, and m.p. 230°C. UV-Vis for **1** (DMF) *λ*/nm (log*ε*): 383 (2.78); 330 sh (3.48); 320 (3.50). IR cm^−1^: 3275 m, 3208 m (*ν*(OH)); 3147 s, 3080 m, 2920 (*ν*(NH_2_, NH)); 1664 (*ν*(C=O)), 1583 s (*ν*(C=N)); 1322 (*ν*(C–O)); 916 s (*ν*(NN)). ^1^H-NMR (DMSO-d_6_): *δ* 12.36 (br, NH), 11.23 (s, C3–OH), 8.05 (d, H4), 7.80 (t, H5), 8.35 (d, H6), 8.14 (s, H7), 6.75 (br, NH_2_); ^13^C-NMR: *δ* 131.4 (C2), 154.0 (C3), 126.4 (C4), 126.4 (C5), 133.70 (C6), 134.7 (C7), 155.9 (C8=O). Anal. calc. for C_7_H_8_N_4_O_2_ (180.0 g mol^−1^): C 46.7, H 4.5, N 31.10; found: C 46.6, H 4.3, N 31.3%.

#### 2.2.2. [Ph_2_(HyFoSc)Sn] (**2**)

Diphenyltin(IV) oxide (0.578 g, 2.0 mmol) and 3-hydroxypyridine-2-carbaldehyde semicarbazone (0.360 g, 2.0 mmol) in benzene (100 mL) were refluxed for 24 h under azeotropic removal of H_2_O (Dean–Stark trap). The resulting clear solution was concentrated *in vacuo *to a small volume. The oily product was chilled and triturated with distilled diethyl ether (Et_2_O) to give a yellow solid. The yellow powder was recrystallized from distilled ether and was dried *in vacuo* over silica gel. Yield 24%; m.p. 209°C. UV-Vis for **2** (DMF) *λ*/nm (log*ε*): 388br (3.87); 331 sh (4.15); 320 (4.18). IR cm^−1^: 3144s, 3065 m, (*ν*(NH_2_)); 1664 (*ν*(C=O)), 1542 s (*ν*(C=N)); 1283 (*ν*(C–O)); 997 s (*ν*(NN)) 339 sh, 321 ms (*ν*(SnC)); 446 sh (*ν*(SnN_C=N_)); 284 mw (*ν*(SnO_C=O_)); 248 mw (*ν*(SnO)). ^1^H-NMR (DMSO-d_6_): *δ* 7.30 (d, H4), 7.36 (t, H5), 8.15 (d, H6), 8.20 (s, H7), 6.74 (br, NH_2_), 7.95 (s, H*o*), 7.45, 7.57 (m, H*m,p*). ^13^C-NMR: *δ* 137.5 (C2), 153.2 (C3), 128.0 (C4), 128.2 (C5), 138.0 (C6), 139.5 (C7), 155.8 (C8=O), 134.7 (C*o*), 127.7 (C*m*), 127.2 (C*p*). Anal. calc. for C_19_H_16_N_4_O_2_Sn (451.0 g mol^−1^) C, 50.6; H, 3.6; N, 12.4; Found: C, 50.4; H, 3.7; N, 12.3%. Crystals suitable for X-ray analysis were obtained by slow evaporation of a freshly distilled diethyl ether solution of **2**.

### 2.3. X-Ray Crystallography

Crystal data are given in Table 1, together with refinement details. All measurements were performed on a Kuma KM4CCD kappa-axis diffractometer with graphite-monochromated MoK*α* radiation (*λ* = 0.71073 Å). The data were corrected for Lorentz and polarization effects. An analytical absorption correction was applied to the data using a multifaceted crystal model [24]. Data reduction and analysis were carried out with the Kuma Diffraction (Wroclaw) programs. The structure was solved by direct-methods and refined by a full-matrix least-squares method on all *F* ^2^ data using the SHELXL97 [25]. Nonhydrogen atoms were refined with anisotropic displacement parameters; all hydrogen atoms were located from different Fourier maps. The C-bound H atoms were refined with the riding model approximation, while the N-bound H-atoms were freely refined isotropically. Molecular graphics were performed with PLATON 2004 [26].

Crystallographic data for **2** have been deposited with the Cambridge Crystallographic Data Centre, CCDC, 634269 for compound **3**. Copies of this information may be obtained free of charge from The Director, CCDC, 12, Union Road, Cambridge CB2 1EZ [FAX +44(1223)336-033] or e-mail mdeposit@ccdc.cam.ac.uk or http://www.ccdc.cam.ac.uk.

### 2.4. Antiproliferative Assay In Vitro

**Compounds**

Test solutions of the tested compounds (1 mg/mL) were prepared by dissolving the substance in 100 *μ*L of DMSO completed with 900 *μ*L of tissue culture medium. Afterwards, the tested compounds were diluted in culture medium to reach the final concentrations of 100, 50, 10, 1, and 0.1 ng/*μ*L. The solvent (DMSO) in the highest concentration used in the test did not reveal any cytotoxic activity.

**Cells**

The cell lines are maintained in the Cell Culture Collection of the University of Ioannina. Twenty-four hours before addition of the tested agents, the cells were plated in 96-well plates at a density of 10^4^ cells per well. The MCF-7 cells were cultured in the D-MEM (Modified Eagle's Medium) medium supplemented with 1% antibiotic and 10% fetal calf serum. L-929 cells were grown in Hepes-buffered RPMI 1640 medium supplemented with 10% fetal calf serum, penicillin (50 U/mL), and streptomycin (50 mg/mL). A-549 cells were grown in F-12K Ham's medium supplemented with 1% glutamine, 1% antibiotic/antimycotic, 2% NaHCO_3_, and 10% fetal calf serum. The cell cultures were maintained at 37°C in a humid atmosphere saturated with 5% CO_2_. Cell numbers were counted by the Trypan blue dye exclusion method. MCF-7, L-929, and A-549 cells were determined by the sulforhodamine B assay [27], while T24 cells by the MTT assay [28].The in vitro tests were performed as described previously [29].

## 3. Results and Discussion

### 3.1. Synthesis

**Scheme 1:**
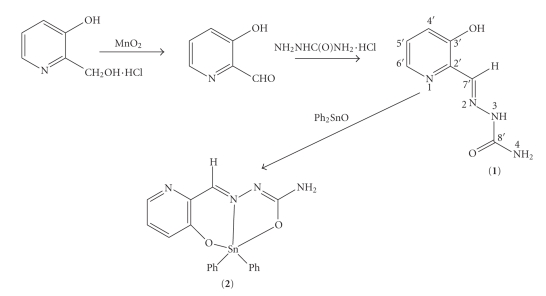
The reaction scheme for synthesis of **1 **and** 2.**

Compound **1 **was synthesized by means of the Heinert–Martell reaction (Scheme 1) [22]. The corresponding diorganotin compound **2 **was prepared by reacting diorganotin(IV) oxide with the semicarbazone in benzene solution in a 1 : 1 molar ratio.

### 3.2. Crystal Structure of **2**

**Figure 1:**
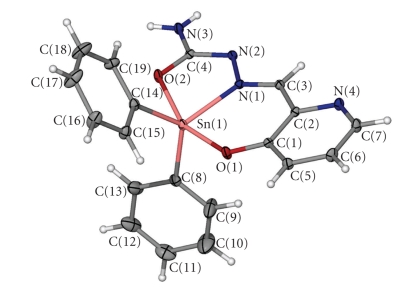
Molecular structure of the diorganotin complex** 2**. Thermal ellipsoids are drawn at the 40% probability level.

A perspective view of **2**, together with the atom-labelling scheme, is given in Figure 1 and selected bond lengths and angles are given in Table 2.

The doubly deprotonated ligand is coordinated to the tin atom through the enolic-oxygen, azomethine-nitrogen, and phenolic-oxygen atoms. Two carbon atoms complete the fivefold coordination at the diorganotin(IV) fragment. Analysis of the shape determining angles using the approach of Addison et al. [30] yields *τ* = 0.54 (*τ* = 0.0 and 1.0 for *SPY *and *TBPY *geometries, resp.). The metal coordination geometry is therefore described as distorted trigonal bipyramidal with the O(2) and O(1) atoms occupying the apical positions around the tin atom.

The dianionic, tridentate ONO ligand has a *ZEZ* configuration, Figure 1. The coordinated part of the ligand is made of three rings, two chelates Sn(1)O(2)N(1)N(2)C(4) (I) and Sn(1)O(1)N(1)C(1)C(2)C(3) (II) and one heterocyclic ring (III). The dihedral angles between the planes of the rings I and II, I and III are 7.17(7) and 10.32(9), respectively, indicating that the ligand as a whole deviates from planarity. The C(3)–N(1) bond length is 1.298(3) Å and is close to the distance of a double bond (1.28 Å). The deprotonation of the N(2)H group produces a negative charge, which is delocalized in the C(3)–N(1)–N(2)–C(4) moiety. The distortion from the ideal trigonal-bipyramidal configuration is shown by the O(1)–Sn(1)–O(2) angle of 156.78(6)°, which deviates from the ideal value of 180°, due in part to the ligand constraint. All the metal-donor bond distances, Table 2, are similar to other organotin complexes presented in the literature [18–21].

**Figure 2:**
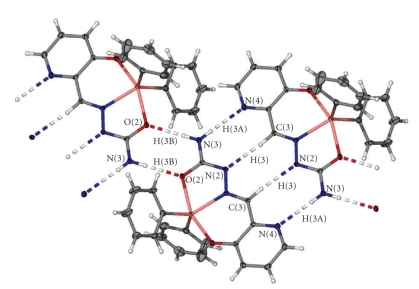
Arrangement of the intermolecular hydrogen bonds in **2. **Thermal ellipsoids are drawn at the 40% probability level.

The polar hydrogen atoms on N(3) participate in two intermolecular hydrogen bonds. The monomers of **2 **are connected into dimers by a pair of cooperative hydrogen bonds [see Table 3 for geometric parameters describing these interactions]. Further, adjacent dimers are connected by C–H ⋯ N contacts, as illustrated in Figure 2. The presence of additional C–H→*π* and *π*→*π* contacts further stabilizes the crystal structure.

### 3.3. Spectroscopic Studies

In the IR spectrum of H_2_HyFoSc (**1**), the strong bands at 3147 and 3088 cm^−1^ are assigned to the asymmetric and symmetric modes of terminal NH_2_, respectively. The *ν*(NH) band appears at 2920 cm^−1^. The strong broad band at *ca*. 2650 cm^−1^ is assigned to the *ν*(NH ⋯ O) and *ν*(OH ⋯ O) mode due to strong intra- or intermolecular hydrogen bonding. The absence of the *ν*(NH) stretching motion at **2 **is indicative of deprotonation of the amide proton. The strong broad band at *ca*. 2650 cm^−1^ at **2 **is probably due to the *ν*(NH ⋯ N) intermolecular hydrogen bonds as confirmed by X-ray crystallography. The coordination of the azomethine-N atom to the tin center was suggested in the IR spectrum by a shift of the *ν*(C=N) band to a lower frequency, along with the occurrence of a *ν*(N–N) band to higher frequency [12, 13, 16]. An IR band at 1322 cm^−1^ for **1** was assigned to *ν*(C–O). This band was found to be shifted to 1283–1289 cm^−1^, in the spectrum of **2**, which indicates the coordination of this O atom. The low energy of the *ν*(C=O) vibration in the spectra of **1**, that is, 1664 cm^−1^, is indicative that the carbonyl O-atom is involved in hydrogen bonding. The replacement of the hydrogen by the metal atom does not shift this band to lower frequency. Coordination of the imine nitrogen is also consistent with the presence of a band at 446 cm^−1^, assignable to *ν*(Sn–N). Bands at 284 and 248 cm^−1^ are assigned to *ν*(Sn–O_c=o_) and *ν*(Sn–O_c-o_), respectively [18–21].

**^1^H- and ^13^C-NMR Spectra**

**Figure 3:**
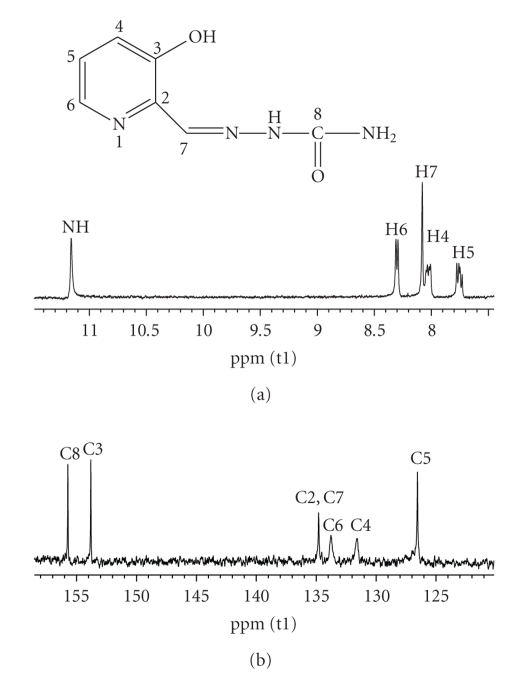
^1^H NMR (a) and ^13^C NMR (b) spectrum of the ligand (**1**).

**Figure 4:**
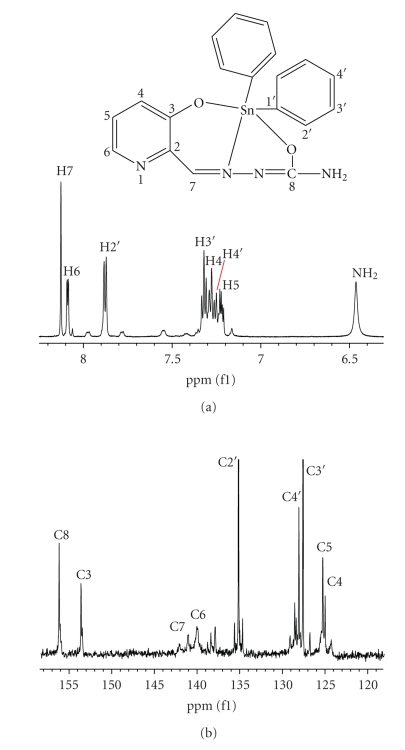
^1^H NMR (a) and ^13^C NMR (b) spectrum of the complex (**2**).

In the ^1^H-NMR spectrum of **1**, the N(3)-H resonance at*δ*12.30 and C–OH at *δ* 11.23 ppm indicates that these H atoms are involved in hydrogen bonding, Figure 3. In the ^1^H-NMR spectrum of **2,** the formyl H-atom H–C(7) was shifted downfield upon coordination, which indicates variations in the electron density at position 7. Deshielding of carbons C4, C5, and C7 is observed in complexes, which is related to the electrophilicity of the tin atom. A *σ*-charge donation from the C–O and N donors to the tin center removes electron density from the ligand and produces this deshielding which will attenuate at positions remote from the tin center, Figure 4.

### 3.4. Pharmacology. Antiproliferative Activity In Vitro

Pd(II) and Pt(II) complexes of 2-carbaldheyde thiosemicarbazone, HFoTsc, were found to be active in vivo against leukemia P388 cells and Pt(II) complexes of N4-ethyl 2-formyl and 2-acetylpyridine thiosemicarbazones showed cytotoxicity and were found to be able to overcome the cisplatin-resistance of A2780/Cp8 cells [10–16]. Also, Zn(II) complexes of 2-carbaldheyde thiosemicarbazone, HFoTsc, and 2-acetylpyridiene thiosemicarbazone, HAcTsc, were found active against the MCF-7 (human breast cancer cell line), T24 (bladder cancer cell line), and a mouse L-929 (a fibroblast-like cell line cloned from strain L) [29]. Compounds **1 **and **2 **and the organotin oxide precursor of **2** were tested for their anti-proliferative activity in vitro against the cells of three human cancer cell lines: MCF-7 (human breast cancer cell line), T24 (bladder cancer cell line), A549 (non-small cell lung carcinoma), and a mouse fibroblast L-929 cell line [see Table 4 for a summary of the cytotoxicity data].

The IC_50_ values for H_2_HyFoSc (**1**) against the MCF-7 and A-549 cell lines are 164 and 175 *μ*M, respectively, while against the L-929 and T-24 cell lines IC_50_ are >555 *μ*M. Thus, **1** is considered as nonactive against these tested cell lines. Compound **2 **is also considered nonactive against T24 cell line. The IC_50_ values for **2** against the L-929 and MCF-7 cell lines are 1.19 and 8.65 *μ*M, respectively. These values are in the same range as observed for cisplatin and Ph_2_SnO, indicating that the observed cytotoxicity is probably due to the cytotoxicity of Ph_2_SnO. Compound **2** may then be considered as a vehicle for activation of the Ph_2_SnO as the cytotoxic agent. The IC_50_ value for **2** against A-549 is 0.086 *μ*M and therefore **2 **is significantly more active compared to Ph_2_SnO (47.1 *μ*M) and cisplatin (1.53 *μ*M), respectively. Compound **2** is thus 547.7 and 17.8 times more cytotoxic than the Ph_2_SnO and cisplatin, respectively, against this cell line. Thus, **2** exhibits selectivity and is considered as an agent with potential antitumor activity against A-549 tumor cell line and can therefore be a candidate for further stages of screening in vitro and/or in vivo.

## Figures and Tables

**Table 1:** X-ray crystal data and structure refinement for **2**.

Empirical formula	C_19_H_16_N_4_O_2_Sn
Formula weight	451.05
Temperature (K)	100(2)
Crystal system	Monoclinic
Space group	*P*2_1_/*n*
Crystal size (mm)	0.17 × 0.20 × 0.23
*a* (Ǻ)	10.5498(6)
*b* (Ǻ)	12.5794(7)
*c* (Ǻ)	13.9477(8)
*β* (°)	103.594(5)
Volume (Ǻ^3^)	1799.2(2)
*Z*	4
*D*_calcd_ (g cm^−3^)	1.665
Absorption coefficient (mm^−1^)	1.441
*θ* range for data collection (°)	3.0–36.6
Reflections collected	29597
Independent reflections (*R*_int_)	8324 (0.056)
Data/parameters	8324/243
Goodness-of-Fit (*F* ^2^)	0.857
Final *R* indices (*I* > 2*σ*(*I*))	0.037
*w* *R*	0.062
Maximum and minimum residuals (e·Ǻ^−3^)	1.03/−0.74

**Table 2:** Selected bond lengths (Å) and angles (°) for complex **2**.

Sn(1)–O(1)	2.073 (2)	O(1)–Sn(1)–O(2)	156.78(6)
Sn(1)–O(2)	2.152(2)	O(1)–Sn(1)–N(1)	84.71(6)
Sn(1)–N(1)	2.166(2)	O(1)–Sn(1)–C(8)	98.51(7)
Sn(1)–C(8)	2.115(2)	O(1)–Sn(1)–C(14)	95.64(7)
Sn(1)–C(14)	2.121(2)	O(2)–Sn(1)–N(1)	73.14(6)
O(1)–C(1)	1.326(2)	O(2)–Sn(1)–C(8)	95.84(7)
O(2)–C(4)	1.297(2)	O(2)–Sn(1)–C(14)	91.13(7)
N(1)–N(2)	1.388(2)	N(1)–Sn(1)–C(8)	110.80(7)
N(1)–C(3)	1.298(3)	N(1)–Sn(1)–C(14)	123.88(7)
N(2)–C(4)	1.329(3)	C(8)–Sn(1)–C(14)	124.44(8)
N(3)–C(4)	1.338(3)	Sn(1)–O(1)–C(1)	132.5(2)
N(4)–C(2)	1.361(3)	Sn(1)–O(2)–C4	113.6(2)
N(4)–C(7)	1.330(3)	Sn(1)–N(1)–N(2)	116.2(2)

**Table 3:** Geometric parameters for hydrogen bonds and for C–H—*π* and *π* ⋯ *π* interactions in **2**.

D	H	A	H ⋯ A	D ⋯ A
N(3) –H(3A) ⋯ N(4)^(i)^		2.29(2)	3.070(3)	175(3)
N(3) –H(3B) ⋯ O(2)^(ii)^		2.07(3)	2.925(2)	183(4)
C(3) –H(3) ⋯ N(2)^(i)^		2.34(2)	3.280(3)	170(3)
C(19) –H(19) ⋯ O(2)		2.51(2)	3.087(3)	119
C–H(I)→Cg(J)^(a)^		H–Cg	C–Cg	∠C–H–Cg
C(7) –H(7)→Cg(5)^(iii)^	2.76	3.619(2)	151	
C(12) –H(12)→Cg(5)^(iv)^	2.86	3.722(3)	152	
Cg(I)→Cg(J)^(a)^	Cg–Cg^(b)^	*β* ^ (c)^	CgI–Perp^(d)^	CgJ–Perp^(e)^
Cg(3)→Cg(4)^(v)^	3.824(2)	10.55	3.7122(8)	3.759(2)
Cg(4)→Cg(3)^(iv)^	3.823(2)	10.55	3.759(2)	3.7121(8)

^(a)^Where Cg(3), Cg(4), and Cg(5) are referred to the centroids N(4)C(1)C(2)C(5)C(6)C(7), C(8)–C(14), and C(14)–C(19); ^(b)^Cg–Cg is the distance between ring centroids; symmetry transformations, (i) 2 − *x*, 1 − *y*, −*z*; (ii) 1 − *x*, 1 − *y*, −*z*; (iii) 1 + *x*, *y*, *z*; (iv) −1/2 + *x*, 1/2 − *y*, −1/2 + *z*; (v) 1/2 + *x*, 1/2 − *y*, 1/2 + *z*; ^(c)^Where *β* is the angle Cg(I)→Cg(J) or Cg(i)→Me vector and normal to plane I (°); ^(d)^CgI–Perp is the perpendicular distance of Cg(I) on ring J; ^(e)^CgJ–Perp is the perpendicular distance of Cg(J) on ring I.

**Table 4:** The antiproliferative activity in vitro of H_2_HyFoSc and its organotin complex **2** (expressed as ID_50_ (*μ*M)) against MCF-7, T-24, A-549, and L-929 cancer cell lines.

Compounds	L-929	A-549	T-24	MCF-7
[H_2_HyFoSc] (**1**)	n.a	175.0	n.a.	164.0
[Ph_2_(HyFoSc)Sn] (**2**)	1.19	0.086	n.a.	8.65
[Ph_2_SnO]	10.73	47.10	n.a	3.46
Cisplatin	0.69	1.53	41.7	8.00
